# Two versus five days of antibiotics after appendectomy for complex acute appendicitis (APPIC): study protocol for a randomized controlled trial

**DOI:** 10.1186/s13063-018-2629-0

**Published:** 2018-05-02

**Authors:** Anne Loes van den Boom, Elisabeth M. L. de Wijkerslooth, Joost van Rosmalen, Frédérique H. Beverdam, Evert-Jan G. Boerma, Marja A. Boermeester, Joanna W. A. M. Bosmans, Thijs A. Burghgraef, Esther C. J. Consten, Imro Dawson, Jan Willem T. Dekker, Marloes Emous, Anna A. W. van Geloven, Peter M. N. Y. H. Go, Luc A. Heijnen, Sander A. Huisman, Dayanara Jean Pierre, Joske de Jonge, Jurian H. Kloeze, Marc A. Koopmanschap, Hester R. Langeveld, Misha D. P. Luyer, Damian C. Melles, Johan W. Mouton, Augustinus P. T. van der Ploeg, Floris B. Poelmann, Jeroen E. H. Ponten, Charles C. van Rossem, Wilhelmina H. Schreurs, Joël Shapiro, Pascal Steenvoorde, Boudewijn R. Toorenvliet, Joost Verhelst, Hendt P. Versteegh, Rene M. H. Wijnen, Bas P. L. Wijnhoven

**Affiliations:** 1 Department of Surgery, Erasmus MC – University Medical Centre Rotterdam, PO Box 2040, 3000 CA Rotterdam, The Netherlands; 2 Department of Biostatistics, Erasmus MC – University Medical Centre, Rotterdam, The Netherlands; 3 Department of Surgery, Franciscus Gasthuis & Vlietland, Rotterdam, The Netherlands; 4 Department of Surgery, Zuyderland MC, Sittard/Heerlen, The Netherlands; 5 Department of Surgery, Academisch Medisch Centrum, Amsterdam, The Netherlands; 6 Department of Surgery, Meander MC, Amersfoort, The Netherlands; 7 Department of Surgery, IJsselland Ziekenhuis, Capelle a/d IJssel, The Netherlands; 8 Department of Surgery, Reinier de Graaf Gasthuis, Delft, The Netherlands; 9 Department of Surgery, MC Leeuwarden, Leeuwarden, The Netherlands; 10 Department of Surgery, Tergooi, Hilversum/Blaricum, The Netherlands; 11 Department of Surgery, St. Antonius Ziekenhuis, Nieuwegein, The Netherlands; 12 Department of Surgery, Noordwest Ziekenhuisgroep, Alkmaar, The Netherlands; 13 Department of Surgery, Medisch Spectrum Twente, Enschede, The Netherlands; 14 Erasmus School of Health Policy and Management, Erasmus University, Rotterdam, The Netherlands; 15 Department of Pediatric Surgery, Erasmus MC – University Medical Centre, Rotterdam, The Netherlands; 16 Department of Surgery, Catharina Ziekenhuis, Eindhoven, The Netherlands; 17 Department of Medical Microbiology and Infectious Diseases, Erasmus MC – University Medical Centre, Rotterdam, The Netherlands; 18 Department of Surgery, Maasstad Ziekenhuis, Rotterdam, The Netherlands; 19 Department of Surgery, Ikazia Ziekenhuis, Rotterdam, The Netherlands

**Keywords:** Acute appendicitis, Complex appendicitis, Antibiotic prophylaxis, .

## Abstract

**Background:**

Acute appendicitis is one of the most common indications for emergency surgery. In patients with a complex appendicitis, prolonged antibiotic prophylaxis is recommended after appendectomy. There is no consensus regarding the optimum duration of antibiotics. Guidelines propose 3 to 7 days of treatment, but shorter courses may be as effective in the prevention of infectious complications. At the same time, the global issue of increasing antimicrobial resistance urges for optimization of antibiotic strategies. The aim of this study is to determine whether a short course (48 h) of postoperative antibiotics is non-inferior to current standard practice of 5 days.

**Methods:**

Patients of 8 years and older undergoing appendectomy for acute complex appendicitis – defined as a gangrenous and/or perforated appendicitis or appendicitis in presence of an abscess – are eligible for inclusion. Immunocompromised or pregnant patients are excluded, as well as patients with a contraindication to the study antibiotics. In total, 1066 patients will be randomly allocated in a 1:1 ratio to the experimental treatment arm (48 h of postoperative intravenously administered (IV) antibiotics) or the control arm (5 days of postoperative IV antibiotics). After discharge from the hospital, patients participate in a productivity-cost-questionnaire at 4 weeks and a standardized telephone follow-up at 90 days after appendectomy. The primary outcome is a composite endpoint of infectious complications, including intra-abdominal abscess (IAA) and surgical site infection (SSI), and mortality within 90 days after appendectomy. Secondary outcomes include IAA, SSI, restart of antibiotics, length of hospital stay (LOS), reoperation, percutaneous drainage, readmission rate, and cost-effectiveness. The non-inferiority margin for the difference in the primary endpoint rate is set at 7.5% (one-sided test at ɑ 0.025). Both per-protocol and intention-to-treat analyses will be performed.

**Discussion:**

This trial will provide evidence on whether 48 h of postoperative antibiotics is non-inferior to a standard course of 5 days of antibiotics. If non-inferiority is established, longer intravenous administration following appendectomy for complex appendicitis can be abandoned, and guidelines need to be adjusted accordingly.

**Trial registration:**

Dutch Trial Register, NTR6128. Registered on 20 December 2016.

**Electronic supplementary material:**

The online version of this article (10.1186/s13063-018-2629-0) contains supplementary material, which is available to authorized users.

## Background

Acute appendicitis is one of the most common surgical emergencies in children and adults worldwide [1–3]. Although the role of surgery as primary treatment has recently been questioned, appendectomy remains the treatment of choice [4, 5]. In the Netherlands, more than 12,000 patients undergo appendectomy for acute appendicitis each year [6]. In Northern America the estimated number of patients with appendicitis in 2015 was over 378,000 [7]. Intraoperatively, acute appendicitis is classified as either simple or complex. A phlegmonous appendix is considered simple. A complex appendicitis includes a gangrenous and/or perforated appendix as well as any appendicitis with an intra-abdominal or pelvic abscess (IAA) [8]. Previously, it was thought that a simple appendicitis could progress towards a complex appendicitis over time, but more recent data suggest that both entities represent distinct types of inflammation [8, 9]. Some 25–30% of all patients with appendicitis have a complex appendicitis, which is associated with increased risk of postoperative infectious complications [10–14]. Therefore, following perioperative antibiotic prophylaxis, guidelines recommend postoperative antibiotics for complex appendicitis [15–18].

Currently, there is no consensus on the duration of postoperative antibiotic treatment and different antibiotic regimens are used [8, 19–21]. A nationwide cohort study from the Netherlands showed that most patients receive 5 days of postoperative antimicrobial therapy [22]. However, it may be safe to stop intravenously administered (IV) antibiotic treatment earlier than 5 days, when a patient meets defined discharge criteria (patient is afebrile, has a normal leukocyte count, has resumed oral intake) [10, 14, 23–29]. Cohort studies show that 3 days of postoperative antibiotic treatment is feasible and safe [12, 30–32]. At least 48 h of IV antibiotics is recommended in the Dutch surgical guideline [15]. Small retrospective studies show that even postoperative prophylaxis of less than 3 days is feasible [33–36]. However, the methodological quality of these studies is poor. Therefore, no definite recommendations can be made regarding the optimum duration of postoperative prophylaxis after appendectomy for complex appendicitis. To date, no randomized clinical trial has been published to address this topic in an adequately powered study population.

Furthermore, there is a growing global health issue of bacterial resistance. Antimicrobial resistance is a natural biological outcome of antibiotic use and antibiotic overtreatment speeds up this process [37]. Hence, restricting antibiotic therapy is warranted, as pointed out in a report by the World Health Organization [38]. This study aims to evaluate efficacy of a restrictive postoperative antibiotic course as compared to standard regimen for complex appendicitis, in a non-inferiority design. This manuscript is prepared in accordance with the Standard Protocol Items: Recommendations for Interventional Trials (SPIRIT) guidelines [39].

### Trial objective and hypothesis

The primary objective of this study is to evaluate the efficacy and safety of discontinuing antibiotic treatment after 48 h, compared to completing a standard course of 5 days after appendectomy for complex acute appendicitis. It is hypothesized that a 48-h course is non-inferior to 5 days and will not result in an increase of infectious complications and mortality. Secondary aims are to evaluate length of hospital stay and cost-effectiveness.

## Methods

### Trial design

**Fig. 1:**
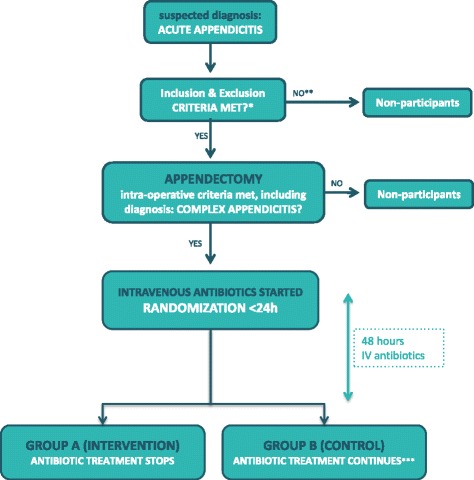
APPIC flowchart of inclusion and randomization. Legend: * All except intraoperative criteria regarding type of appendicitis; ** If the patient has not been able to give informed consent prior to appendectomy, this may still be acquired postoperatively, as long as inclusion and randomization takes place within 24 h; *** Intravenously administered antibiotic treatment continues for three more days to complete 5 days in total

**Fig. 2:**
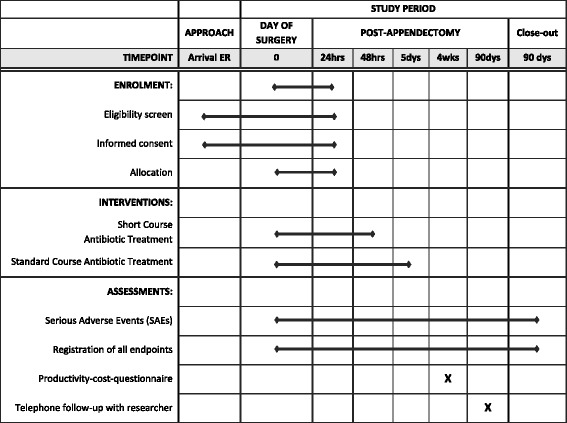
APPIC schedule of enrollment, interventions, and assessments

The *A*ntibiotics following a*PP*endectomy *I*n *C*omplex appendicitis (APPIC) trial is a phase IV, prospective, multicenter, non-blinded, randomized controlled trial powered for non-inferiority. Patients are randomly allocated to a short course of 48 h (intervention arm), or the standard course of 5 days (control arm) of IV antibiotics following appendectomy for complex appendicitis. An overview of enrollment, interventions, and follow-up of participants in the APPIC trial is shown in Fig. 1. Figure 2 shows the Standard Protocol Items Recommendations for Interventional Trials (SPIRIT) Figure. The SPIRIT Checklist is shown in Additional file 1.

### Trial setting

The trial will run in at least 14 hospitals in the Netherlands. This includes one academic hospital and 13 teaching hospitals. The participating hospitals are listed on the trial webpage (www.appictrial.nl). In all participating hospitals appendectomy is mostly performed laparoscopically.

### Eligibility criteria

Patients of at least 8 years of age who are scheduled to undergo surgery for suspected acute appendicitis will be approached for participation in the study. If a complex appendicitis is diagnosed intraoperatively, patients are eligible for inclusion. A complex appendicitis is defined as a gangrenous and/or perforated appendicitis or any appendicitis in presence of an IAA [8]. Written informed consent is preferably obtained before surgery, but may be obtained postoperatively as long as inclusion and randomization is performed within 24 h after surgery. Exclusion criteria are:

- Unable to give informed consent (language barrier, legally incapable)
- Interval appendectomy
- Clinical suspicion of severe sepsis*
- Conservative treatment of acute appendicitis
- American Society of Anesthesiologists (ASA) score IV or not able to undergo surgery
- Known allergy or other contraindication to study medication*
- Immunocompromised patients*
- Pregnancy
- Concurrent use of antibiotics for other indication*
- Simple acute appendicitis*
- Appendicular infiltrate not amendable for appendectomy
- Inadequate source control in opinion of the surgeon*

* more elaborate definitions are given in the full study protocol.

### Interventions

#### Postoperative antibiotic treatment

Participants will be randomized (1:1) to receive either: (1) a short course of 48 h or (2) a standard 5-day course of postoperative antibiotic treatment. All patients receive IV antibiotics during the first 48 h after appendectomy: cefuroxime/metronidazole (three times a day, 1500/500 mg), or alternatively ceftriaxone/metronidazole (once a day, 2000 mg/three times a day, 500 mg) according to local antibiotic policy. In the control group the IV antibiotics are continued for three more days (a switch to an oral formula is not allowed). A daily dose of gentamicin as co-intervention is optional. No other antibiotics are permitted.

#### Criteria for modifying the allocated treatment

Antibiotic treatment may be prolonged or restarted only in case of a proven source of infection (a decision algorithm is provided in the full protocol). A switch to a different antibiotic regimen is allowed only if necessary due to an adverse reaction to the antibiotics or if indicated by culture results (if a micro-organism resistant to cefuroxime (or ceftriaxone) is cultured a switch should be made to ensure effective antibiotic treatment).

#### Discharge and follow-up

Laboratory tests, imaging studies, and blood cultures will be performed only when clinically indicated. The following clinical parameters will be registered on a daily basis: body temperature < 38° Celsius, able to tolerate oral intake, able to mobilize independently; Visual Analog Scale (VAS) < 4 requiring only orally administered analgesia. However, these criteria are not mandatory for discharge and ultimately the responsible physician decides when a patient is able to go home. After discharge a standard outpatient visit is planned at 2 to 4 weeks according to local hospital policy. Four weeks after appendectomy, patients are asked to complete a productivity-cost questionnaire. At 90 days after appendectomy a standardized follow-up by telephone will be conducted.

### Outcome measures

All outcome measures will be registered directly from the electronic patient files. Outcome assessors will not be blinded for the treatment allocation. The telephone follow-up is introduced to check missing data on the primary endpoint; e.g., visits to hospitals or medical facilities other than the center where the patient was treated and included into the trial.

#### Primary outcome measure

The primary endpoint of this trial is a composite endpoint of infectious complications related to appendectomy, including IAA and surgical site infection (SSI), and mortality within 90 days after appendectomy. An IAA is defined as an infection that involves the abdominal part of the body deeper than the fascial/muscle layers that is opened or manipulated during the operative procedure. IAA can be diagnosed through imaging or during reintervention, through purulent drainage from a drain placed into the IAA, or isolation of organisms from a culture of the IAA [40]. An SSI can be either deep or superficial, involving the skin, subcutaneous tissue and/or deep soft tissues of the incision. IAA and SSI are defined in more detail according to the Center for Disease Control (CDC) criteria in the full study protocol [40].

#### Secondary outcome measures

Secondary endpoints are separate rates of IAA, SSI and mortality; duration of antibiotic treatment; the antibiotic regimen; proportion of patients that restarted antibiotics; length of hospital stay (LOS); time to fulfill discharge criteria; postoperative complications; reoperation; percutaneous drainage; number of visits to the general practitioner (GP), emergency room (ER) and outpatient clinic; readmission rate; adverse events on antibiotics; and cost-effectiveness. Complications will be classified according to the Clavien-Dindo classification of surgical complications as well as the Comprehensive Complication Index (CCI). To analyze cost-effectiveness, the validated Institute for Medical Technological Assessment – Productivity Cost Questionnaire (iMTA-PCQ) (version October 2012) will be used, enhanced with a section concerning school absence.

### Sample size calculation

A power analysis was performed based on a one-sided 97.5% confidence interval for the effect of study arm (intervention or control), an expected 15% primary endpoint rate and a 7.5% non-inferiority margin. To obtain a power of 90%, 960 patients are needed (480 per treatment arm). To account for possible effects of dropout and missing data (10%) we will recruit 1066 patients. This sample size should also yield sufficient power for the analysis of secondary endpoints.

### Recruitment

Recruitment of participants started on 12 April 2017 and is ongoing. Additional participating hospitals may be recruited to ensure feasibility of the trial. The target of 1066 patients is expected to be completed in early 2020.

### Allocation

Computerized block randomization (stratified for center) will take place within 24 h after surgery through ALEA, a web-based application managed by the Clinical Trial Center (CTC) of the Erasmus MC. Random blocks of different lengths are used. Eligible patients will be randomized in a 1:1 ratio to arm A (short course) or arm B (standard course). Each patient will be given a unique study number. An independent data manager from the CTC who is not involved with the clinical practice or patient recruiting created the randomization sequence. The result of the randomization and the patient study number will immediately be provided through ALEA per email to all parties predefined in the system who should receive such notifications.

### Implementation

Before the start of the trial, each center is visited by the research team to inform and instruct the involved personnel on study-specific procedures. Surgeons and residents are trained how to assess the type of appendicitis to decide whether patients are eligible for study participation by means of recorded examples of all types of appendicitis.

### Blinding

Blinding for treatment allocation in this study would not only be difficult to achieve, but is also undesirable because good clinical decision-making during the postoperative course requires specific knowledge of antibiotics that have or have not been given to the patient. Therefore, this is an non-blinded trial.

### Data collection and management

A data manager from each participating hospital will carry out the data collection in collaboration with the trial coordinator. Baseline demographics, as well as preoperative, intraoperative and postoperative variables, will be collected from the electronic medical records. The validated iMTA-PCQ will be used for cost analysis. A list of all variables is provided in the full study protocol. All data will be entered into the secure online ALEA database, a system validated and supported by the Erasmus University Medical Centre. Data will be handled confidentially and anonymously. A short intraoperative video or static picture(s) should be recorded for quality assurance of the diagnosis complex appendicitis. Quality control will involve collecting data on adherence to the intervention, patient inclusion and follow-up, as well as monitoring the quality of the data entry. Qualified data managers of the CTC of the Erasmus MC will perform quality control and assurance. Checks and queries will be performed to ensure quality, consistency, and completeness. Missing data and inconsistencies will be reported back to the centers to be clarified by the local responsible investigator.

### Statistical analysis

We anticipate a 15% rate of infectious complications and mortality in this study population. A 7.5% difference (non-inferiority margin) in the primary endpoint rate is deemed acceptable between the intervention group and control group. This margin is considered acceptable since mortality is expected to account for a negligible proportion within the primary endpoint and infectious complications after appendectomy can be well treated with minimum morbidity and long-term consequences.

#### Primary endpoint

The study hypothesis will be tested by a one-sided 97.5% confidence interval for the effect of study group (absolute risk difference). This confidence interval will be adjusted for effects of type of appendicitis and age (as a single categorical covariate: < 16 years old/non-perforated, < 16 years old/perforated, ≥16 years old/non-perforated, ≥16 years old/perforated) using the method proposed by Klingenberg [41, 42]. Non-inferiority will be established if the upper limit of the confidence interval is lower than 7.5%. Both per-protocol and intention-to-treat analyses will be performed. In a secondary analysis, logistic regression analysis will be performed to identify predictors of the composite primary endpoint. Independent variables in this model will include treatment group and also age, sex, surgical approach, type of appendicitis, ASA score, and center, as well as significant interaction effects of these independent variables with treatment group.

#### Secondary endpoints

General patient characteristics and other clinically relevant parameters will be compared between the intervention group and the control group with the independent samples Student’s *t* test or the Mann-Whitney test in case of continuous outcome variables and the chi-square or Fisher’s exact test in case of categorical outcome variables where appropriate. All secondary endpoints will be compared between the trial arms using linear regression for continuous outcomes and logistic regression for dichotomous outcomes, with adjustment for age, sex, surgical approach (open versus laparoscopic), type of appendicitis, ASA score, and center. In case of non-normally distributed continuous outcomes, appropriate transformation of these outcomes will be applied. A two-sided significance level of 0.05 will be used for all secondary analyses. Uncertainty with respect to cost-effectiveness will be analyzed by bootstrapping results for incremental costs and health effects. The results will be shown in an acceptability curve that indicates the probability that the intervention meets several cost-effectiveness thresholds.

### Data monitoring and safety

An independent safety committee (DSMB) is assembled to monitor trial safety and progress, with special focus on imbalance between the two trial arms in 90-day mortality and serious postoperative complications. The DSMB is composed of a statistician, two surgeons and a microbiologist, all of whom are unrelated to the study and have no conflict of interest with the coordinating investigator of the study. There will be two planned formal safety analyses: after the first 266 included patients have completed follow-up and after 666 patients have completed follow-up. Safety stopping rules will be applied using the alpha spending approach of O’Brien and Fleming, described into more detail in the full study protocol. The DSMB will notify the coordinating and principal investigators if conditions of the stopping rules have been reached. The Steering Committee will decide on continuation of the trial. The DSMB roles, responsibilities, meetings and logistics are outlined in the APPIC trial DSMB Charter.

Independent monitors of the CTC of Erasmus MC will visit participating centers intervals at regular intervals to verify adherence to the protocol and legal requirements and perform source data verification. A first site monitoring visit will take place at each participating hospital after the first three randomized patients have completed follow-up. Subsequent monitoring visits will be planned according to the predefined monitoring plan.

### Rationale for the chosen study design

A non-inferiority design is chosen as the objective of this trial is to show that a short course of antibiotics is *no less effective* than a standard course, in terms of preventing infectious complications. This is relevant in light of several potential advantages of reduced use of antibiotics, such as fewer adverse reactions to antibiotics, shorter length of hospital stay, lower medical care costs and less antimicrobial resistance. In the academic literature, postoperative infectious complications are reported in 15–20% of patients [43–45]. Furthermore, a similar study by Sawyer et al. was aimed at detecting a 10% difference in complication rates after a shorter course of postoperative antibiotic treatment in complicated intra-abdominal infections [28]. Based on these findings and the fact that a reduction in antibiotic consumption will lead to a significant reduction in costs and antimicrobial resistance, we accept a 7.5% difference (non-inferiority margin) in the primary endpoint rate. A non-inferiority trial with this margin is acceptable based on the assumption that infectious complications after an appendectomy for a complex appendicitis are in general not associated with severe morbidity and/or mortality. Since it is known that treatment with IV antibiotics for 48 h ensures adequate tissue concentrations (to eliminate the relevant micro-organisms such as *E. coli*) [46–48], we have chosen 48 h of IV antibiotics as our intervention. For the individual patient advancing from the regular (3 to) 5 days of antibiotics towards 48 h may not seem an enormous step forward. However, extrapolating this to all patients with complex appendicitis could have a major impact on healthcare. From a methodological perspective, we choose to administrate antibiotics completely intravenously for both the intervention and the control group. Some studies found no support for use of orally administered antibiotics after the initial postoperative intravenous administration [26, 49]. In addition, it is questioned if adequate tissue concentrations can be met by orally administered antibiotics for bacteria commonly isolated in complex appendicitis [50]. Complete intravenous courses will ensure homogenous treatment in both study arms, without patients’ compliance or effectiveness of orally administered antibiotics as uncertainties.

## Discussion

The present study is designed to answer the question whether 48 h of postoperative antibiotics is non-inferior to the standard treatment of 5 days in patients with a complex appendicitis. If non-inferiority is established, this may lead to a reduction in the use of antibiotics in the future. This in turn may shorten length of hospital stay and may result in lower hospital costs. In the longer term, less use of antibiotics may slow down emergence of antimicrobial resistance.

One of the five main objectives in the global action plan on antimicrobial resistance by the World Health Organization (WHO) is “to optimize the use of antimicrobial medicines” [51]. The global threat of antimicrobial resistance urges for action against overuse. More research is needed to determine the minimum effective courses for many diseases. For several infections (e.g., pneumonia, pyelonephritis, cellulitis) shorter courses have proven just as effective as extended courses [52]. Yet, for many diseases, including appendicitis, proper studies have not been performed [53]. With a lifetime risk of about 7 to 8% and a pooled incidence of 100 to 151 per 100,000 person-years in the Western World, acute appendicitis is one of the most common surgical emergencies worldwide [1, 7, 8]. The 25 to 30% of complex appendicitis represents a substantial number of patients who receive prolonged antibiotic prophylaxis, as recommended by the guidelines [15, 16, 18]. To date, no randomized study has evaluated a reduced course of postoperative antibiotics in an adequately powered study. Some studies – all including pediatric patients – have compared a course with a predefined minimum duration (mostly 4 days) with a variable duration based on clinical and laboratory parameters (body temperature < 38 °C, resumed oral intake, white blood cell count) [14, 23–25, 32]. However, these clinical parameters may still cause overtreatment with antibiotics, as an increased body temperature or delayed clinical improvement may well reflect a prolonged sterile SIRS response rather than an infectious focus [54]. Median antibiotic treatment duration was still 5 days in most studies. Evidence for restricting postoperative antibiotics to less than 3 days after appendectomy is limited. Two retrospective studies demonstrated that antibiotics for more than 24 h after surgery for complex appendicitis does not reduce the rate of infectious complications. Kimbrell et al. [33] included eight patients that had received antibiotics for 24 h at most and 44 patients that had received antibiotics for more than 24 h. Reported IAA rates were 25% and 20.5%, respectively (*p* = 1.00). In a larger study (*n* = 410) by Kim et al. [35] multivariable regression analysis revealed no difference in SSI rate between patients with complex appendicitis that received postoperative prophylaxis (for a median of 7 days (range 2–21)) and patients that did not. Unfortunately, IAA rate was not reported in this study. Two more studies reported interesting results of antibiotic treatment restricted to less than three postoperative days: no intra-abdominal abscesses occurred in 55 and 11 patients that received antibiotics for 24–48 h and 0–24 h, respectively [34, 36]. The small sample sizes and retrospective nature of these studies must be recognized when interpreting the results. Surgeons may be less inclined to prolong prophylaxis in healthier patients and more so in patients that are at increased risk of complications.

Whereas evidence about the duration of postoperative antibiotics for complex appendicitis is missing, this has been evaluated in patients with intra-abdominal infections. The STOP-IT trial investigated a restricted antibiotic course after adequate source-control procedures for complicated intra-abdominal infections [28]. Some 14% of included patients had a complex appendicitis. After a median duration of 4 days of antibiotics in the intervention arm and 7 days in the control arm, infectious complications occurred in 21.8% and 22.3% of the groups, respectively (*p* = 0.92). Some critical notes can be made. Premature closure of the study, due to concerns of futility led to an underpowered study to demonstrate equivalence of both regimens. Also, in a large proportion of patients (23%) the protocol-specified treatment duration was not adhered to [55]. On the other hand, both intention-to-treat and per-protocol analyses were performed and the rate of complications above 20% in both groups confirms that antibiotics may not have a significant role in prevention of infectious complications at all [56].

More recently the PEANUTS trial was published: a multicenter randomized controlled trial of extended (3 days) versus single-dose antibiotic prophylaxis for (mild) acute calculous cholecystitis [57]. Similar rates of postoperative infectious complications were seen in both groups (4%). As for complex appendicitis, the recommended duration of antimicrobial therapy varies in guidelines and there is a lack of randomized trials. In line with results from the STOP-IT trial, no benefit was found for extending postoperative prophylaxis, in a randomized setting. Subsequently, the PEANUTS-II trial started (Dutch Trial Register no. NTR5802), in which patients with (mild) acute calculous cholecystitis are randomized to single-dose perioperative prophylaxis or no antibiotic prophylaxis at all.

A nationwide prospective cohort study from the Netherlands in 2014 showed that in most patients (78%) antibiotics were given for 5 days or more after surgery for complex appendicitis. The authors concluded that 3 days of antibiotics led to a similar rate of infectious complications. Surgical site infections and intra-abdominal abscesses were seen in 1.3% and 1.6% (*p* = 0.89) and 8.0% and 8.9% of patients (*p* = 0.81), respectively [30]. In Denmark, postoperative prophylaxis of 3 days has become standard care already [58]. Moreover, in several hospitals in the UK 24 h (three doses) of antibiotics has been introduced.

Two limitations of this study should be mentioned. Firstly, the present study is non-blinded. Blinding for treatment allocation would require patients in arm A (48 h) to remain admitted to the hospital and receive a placebo drug intravenously for 3 days. This would put a significant strain on length of hospital stay and costs for the participating hospitals. More importantly, in terms of good clinical decision-making it is important for the treating physician to know whether or not the patient is still receiving actual antibiotics. It is important to reduce risk of bias wherever possible, yet blinding in this trial would not be feasible or desirable. Another limitation is the diagnosis of complex appendicitis which can be rather subjective and dependent on individual surgeons’ opinions [59]. As we strived for this trial to follow clinical practice, we chose to keep the definition of complex appendicitis simple (*a gangrenous and/or perforated appendicitis or appendicitis in presence of intra-abdominal abscess*) and to rely on the surgeon’s intraoperative judgement. For quality assurance, a static image or video of the appendicitis is taken for patients included in the APPIC trial. This way, we will be able to assess the reliability and reproducibility of the diagnosis afterwards.

## Trial status

Trial registries: EudraCT 2016–003428-21, issued on 16 August 2016. Dutch trial register (NTR) no. 6128, registered on 20 December 2016. The first investigators’ meeting took place on 3 April 2017. Twelve centers have been initiated and are actively recruiting. The first patient was included on 9 June 2017. In total, 165 patients were randomized, while this manuscript was being completed. Recruitment is expected to end in early 2020.

## Additional files

Additional file 1: Standard Protocol Items: Recommendations for Interventional Trials **(**SPIRIT) Checklist APPIC trial. (PDF 129 kb)

Additional file 2: Subject information and consent form (in Dutch). (PDF 231 kb)

## Abbreviations

APPIC: Antibiotics following aPPendectomy In Complex appendicitis
ASA: American Society of Anesthesiologists
CCI: Comprehensive Complication Index
CDC: Center for Disease Control
CTC: Clinical Trial Center
DSMB: Data Safety Monitoring Board
ER: Emergency room
GP: General practitioner
IAA: Intra-abdominal (or pelvic) abscess
iMTA-PCQ: Institute for Medical Technological Assessment – Productivity Cost Questionnaire
LOS: Length of hospital stay
SPIRIT: Standard Protocol Items: Recommendations for Interventional Trials
SSI: Surgical site infection
VAS: Visual Analog Scale
WHO: World Health Organization

## References

[1] Addiss DG, Shaffer N, Fowler BS, Tauxe RV (1990). The epidemiology of appendicitis and appendectomy in the United States. Am J Epidemiol.

[2] Ohmann C, Franke C, Kraemer M, Yang Q (2002). Status report on epidemiology of acute appendicitis. Chirurg.

[3] Stewart B, Khanduri P, McCord C, Ohene-Yeboah M, Uranues S, Vega Rivera F (2014). Global disease burden of conditions requiring emergency surgery. Br J Surg.

[4] Vons C, Barry C, Maitre S, Pautrat K, Leconte M, Costaglioli B (2011). Amoxicillin plus clavulanic acid versus appendicectomy for treatment of acute uncomplicated appendicitis: an open-label, non-inferiority, randomised controlled trial. Lancet.

[5] Salminen P, Paajanen H, Rautio T, Nordstrom P, Aarnio M, Rantanen T (2015). Antibiotic therapy vs appendectomy for treatment of uncomplicated acute appendicitis: The APPAC Randomized Clinical Trial. JAMA.

[6] National hospital data, managed by the Dutch Healthcare Authority, available from www.opendisdata.nl

[7] Ferris M, Quan S, Kaplan BS, Molodecky N, Ball CG, Chernoff GW (2017). The global incidence of appendicitis: a systematic review of population-based studies. Ann Surg.

[8] Bhangu A, Soreide K, Di Saverio S, Assarsson JH, Drake FT (2015). Acute appendicitis: modern understanding of pathogenesis, diagnosis, and management. Lancet.

[9] Mason RJ (2008). Surgery for appendicitis: is it necessary?. Surg Infect.

[10] Emil S, Elkady S, Shbat L, Youssef F, Baird R, Laberge JM, et al. Determinants of postoperative abscess occurrence and percutaneous drainage in children with perforated appendicitis. Pediatr Surg Int. 2014;30(12):1265–71. 10.1007/s00383-014-3617-4. 10.1007/s00383-014-3617-4 25362478

[11] Cheong LH, Emil S (2014). Outcomes of pediatric appendicitis: an international comparison of the United States and Canada. JAMA Surg..

[12] Van Rossem CC, Schreinemacher MHF, Treskes K, Van Hogezand RM, Van Geloven AAW (2014). Duration of antibiotic treatment after appendicectomy for acute complicated appendicitis. Br J Surg.

[13] St. Peter SD, Sharp SW, Holcomb IGW, Ostlie DJ (2008). An evidence-based definition for perforated appendicitis derived from a prospective randomized trial. J Pediatr Surg.

[14] van Wijck K, de Jong JR, van Heurn LW, van der Zee DC (2010). Prolonged antibiotic treatment does not prevent intra-abdominal abscesses in perforated appendicitis. World J Surg.

[15] (NVvH) DAoSiDNVvH (2010). Richtlijn voor diagnostiek en behandeling van acute appendicitis (Dutch guideline on acute appendicitis diagnostics and treatment).

[16] Mazuski JE, Tessier JM, May AK, Sawyer RG, Nadler EP, Rosengart MR (2017). The Surgical Infection Society Revised Guidelines on the Management of Intra-Abdominal Infection. Surg Infect.

[17] (SWAB) DWPoAPiDSWA (2015). Antibiotic guideline on secondary peritonitis.

[18] Di Saverio S, Birindelli A, Kelly MD, Catena F, Weber DG, Sartelli M (2016). WSES Jerusalem guidelines for diagnosis and treatment of acute appendicitis Review. World J Emerg Surg.

[19] Gorter RR, Eker HH, Gorter-Stam MA, Abis GS, Acharya A, Ankersmit M, et al. Diagnosis and management of acute appendicitis. EAES consensus development conference 2015. Surg Endosc. 2016;30(11):4668–4690. 10.1007/s00464-016-5245-7 PMC5082605 27660247

[20] Chen C, Botelho C, Cooper A, Hibberd P, Parsons SK (2003). Current practice patterns in the treatment of perforated appendicitis in children. J Am Coll Surg.

[21] Giesen LJ, van den Boom AL, van Rossem CC, den Hoed PT, Wijnhoven BP (2016). Retrospective multicenter study on risk factors for surgical site infections after appendectomy for acute appendicitis. Dig Surg.

[22] van Rossem CC, Bolmers MD, Schreinemacher MH, van Geloven AA, Bemelman WA, Snapshot Appendicitis Collaborative Study Group (2016). Prospective nationwide outcome audit of surgery for suspected acute appendicitis. Br J Surg.

[23] Fraser JD, Aguayo P, Leys CM, Keckler SJ, Newland JG, Sharp SW (2010). A complete course of intravenous antibiotics vs a combination of intravenous and oral antibiotics for perforated appendicitis in children: a prospective, randomized trial. J Pediatr Surg.

[24] Desai AA, Alemayehu H, Holcomb GW 3rd, St Peter SD. Safety of a new protocol decreasing antibiotic utilization after laparoscopic appendectomy for perforated appendicitis in children: a prospective observational study. J Pediatr Surg. 2015;50(6):912–4. 10.1016/j.jpedsurg.2015.03.006. 10.1016/j.jpedsurg.2015.03.006 25812441

[25] Yu TC, Hamill JK, Evans SM, Price NR, Morreau PN, Upadhyay VA (2014). Duration of postoperative intravenous antibiotics in childhood complicated appendicitis: a propensity score-matched comparison study. Eur J Pediatr Surg.

[26] Daskalakis K, Juhlin C, Pahlman L (2014). The use of pre- or postoperative antibiotics in surgery for appendicitis: a systematic review. Scand J Surg.

[27] Taylor E, Dev V, Shah D, Festekjian J, Gaw F (2000). Complicated appendicitis: is there a minimum intravenous antibiotic requirement? A prospective randomized trial. Am Surg.

[28] Sawyer RG, Claridge JA, Nathens AB, Rotstein OD, Duane TM, Evans HL (2015). Trial of short-course antimicrobial therapy for intraabdominal infection. N Engl J Med.

[29] Basoli A, Chirletti P, Cirino E, D'Ovidio NG, Doglietto GB, Giglio D (2008). A prospective, double-blind, multicenter, randomized trial comparing ertapenem 3 vs >or=5 days in community-acquired intraabdominal infection. J Gastrointest Surg.

[30] van Rossem CC, Schreinemacher MH, van Geloven AA, Bemelman WA, Snapshot Appendicitis Collaborative Study Group (2016). Antibiotic duration after laparoscopic appendectomy for acute complicated appendicitis. JAMA Surg.

[31] Henry MCW, Walker A, Silverman BL, Gollin G, Islam S, Sylvester K (2007). Risk factors for the development of abdominal abscess following operation for perforated appendicitis in children: a multicenter case-control study. Arch Surg.

[32] Skarda DE, Schall K, Rollins M, Andrews S, Olson J, Greene T (2014). Response-based therapy for ruptured appendicitis reduces resource utilization. J Pediatr Surg.

[33] Kimbrell AR, Novosel TJ, Collins JN, Weireter LJ, Terzian HW, Adams RT (2014). Do postoperative antibiotics prevent abscess formation in complicated appendicitis?. Am Surg.

[34] Hughes MJ, Harrison E, Paterson-Brown S (2013). Post-operative antibiotics after appendectomy and post-operative abscess development: a retrospective analysis. Surg Infect.

[35] Kim DY, Nassiri N, Saltzman DJ, Ferebee MP, Macqueen IT, Hamilton C (2015). Postoperative antibiotics are not associated with decreased wound complications among patients undergoing appendectomy for complicated appendicitis. Am J Surg.

[36] Cho J, Park I, Lee D, Sung K, Baek J, Lee J (2015). Risk factors for postoperative intra-abdominal abscess after laparoscopic appendectomy: analysis for consecutive 1,817 experiences. Dig Surg.

[37] Goossens H (2009). Antibiotic consumption and link to resistance. Clin Microbiol Infect.

[38] World Health Organization. Antimicrobial resistance: global report on surveillance 2014 Available from: http://apps.who.int/iris/bitstream/10665/112642/1/9789241564748_eng.pdf.

[39] Chan AW, Tetzlaff JM, Altman DG, Dickersin K, Moher D (2013). SPIRIT 2013: new guidance for content of clinical trial protocols. Lancet.

[40] Horan TC, Andrus M, Dudeck MA (2008). CDC/NHSN surveillance definition of health care-associated infection and criteria for specific types of infections in the acute care setting. Am J Infect Control.

[41] Mohamed K, Embleton A, Cuffe RL (2011). Adjusting for covariates in non-inferiority studies with margins defined as risk differences. Pharm Stat.

[42] Klingenberg B (2014). A new and improved confidence interval for the Mantel-Haenszel risk difference. Stat Med.

[43] St Peter SD, Adibe OO, Iqbal CW, Fike FB, Sharp SW, Juang D (2012). Irrigation versus suction alone during laparoscopic appendectomy for perforated appendicitis: a prospective randomized trial. Ann Surg.

[44] Fallon SC, Hassan SF, Larimer EL, Rodriguez JR, Brandt ML, Wesson DE (2013). Modification of an evidence-based protocol for advanced appendicitis in children. J Surg Res.

[45] van den Boom AL, Gorter RR, van Haard PM, Doornebosch PG, Heij HA, Dawson I (2015). The impact of disease severity, age and surgical approach on the outcome of acute appendicitis in children. Pediatr Surg Int.

[46] van den Bosch CMGS, Natsch S, Prins JM, Hulscher ME (2015). Quality indicators to measure appropriate antibiotic use in hospitalized adults. Clin Infect Dis.

[47] Cyriac JM, James E (2014). Switch over from intravenous to oral therapy: a concise overview. J Pharmacol Pharmacother.

[48] Sevinc F, Prins JM, Koopmans RP, Langendijk PN, Dankert J, Speelman P (1999). Early change from intravenous to oral antibiotics: “switch therapy” Vroege omzetting van intraveneuze naar orale antibiotica: ‘switchtherapie’. Ned Tijdschr Geneeskd.

[49] Desai AA, Alemayehu H, Holcomb GW, St Peter SD (2015). Safety of a new protocol decreasing antibiotic utilization after laparoscopic appendectomy for perforated appendicitis in children: a prospective observational study. J Pediatr Surg.

[50] de Velde F, de Winter BC, Koch BC, van Gelder T, Mouton JW, Consortium C-N (2016). Non-linear absorption pharmacokinetics of amoxicillin: consequences for dosing regimens and clinical breakpoints. J Antimicrob Chemother.

[51] (WHO) WHO (2015). Global action plan on antimicrobial resistance 2015.

[52] Spellberg B (2016). The new antibiotic mantra—“shorter is better”. JAMA Intern Med.

[53] Llewelyn MJ, Fitzpatrick JM, Darwin E, SarahTonkin C, Gorton C, Paul J (2017). The antibiotic course has had its day. BMJ.

[54] Singer M, Deutschman CS, Seymour CW, Shankar-Hari M, Annane D, Bauer M (2016). The Third International Consensus Definitions for Sepsis and Septic Shock (Sepsis-3). JAMA.

[55] Boermeester M (2015). Kortdurend antibiotica bij intra-abdominale infectie?. Ned Tijdschr Geneeskd.

[56] Zakrison TL (2015). Short-course antimicrobial therapy may be clinically similar to a longer course for complicated intra-abdominal infections. Evid Based Med.

[57] Loozen CS, Kortram K, Kornmann VN, van Ramshorst B, Vlaminckx B, Knibbe CA (2017). Randomized clinical trial of extended versus single-dose perioperative antibiotic prophylaxis for acute calculous cholecystitis. Br J Surg.

[58] Kleif J, Rasmussen L, Fonnes S, Tibaek P, Daoud A, Lund H, et al. Enteral antibiotics are non-inferior to intravenous antibiotics after complicated appendicitis in adults: a retrospective multicentre non-inferiority study. World J Surg. 2017;41(11):2706–2714. 10.1007/s00268-017-4076-6. 10.1007/s00268-017-4076-6 28600695

[59] Ponsky TA, Hafi M, Heiss K, Dinsmore J, Newman KD, Gilbert J (2009). Interobserver variation in the assessment of appendiceal perforation. J Laparoendosc Adv Surg Tech A.

