# Supplementary material for: Two versus five days of antibiotics after appendectomy for complex acute appendicitis (APPIC): study protocol for a randomized controlled trial
Source: Trials. 2018 May 2;19:263. doi: 10.1186/s13063-018-2629-0 (PMC5932884; doi:10.1186/s13063-018-2629-0)
Supplement: Supplementary file 2 — Subject information and consent form (in Dutch). (PDF 231 kb) [file 13063_2018_2629_MOESM2_ESM.pdf]

Proefpersoneninformatie voor deelname aan medisch-wetenschappelijk onderzoek

Onderzoek naar effectiviteit van een kortdurende behandeling met postoperatieve antibiotica bij complexe blindedarmontsteking

Prospectieve, gerandomiseerde multicenter studie waarbij twee dagen antibiotica vergeleken wordt met vijf dagen antibiotica na een operatie voor complexe acute appendicitis.

Inleiding

Geachte heer/mevrouw,

Wij vragen u om mee te doen aan een medisch-wetenschappelijk onderzoek. Meedoen is vrijwillig. Om mee te doen is wel uw schriftelijke toestemming nodig. U ontvangt deze brief omdat u korte termijn een operatie zult ondergaan (of kort geleden hébt ondergaan) ter verwijdering van de blindedarm (“appendectomie”), vanwege een acute blindedarmontsteking (“appendicitis”). Het kan zijn dat de ontsteking tijdens de operatie beoordeeld wordt als complex, waarop u nog een nabehandeling zal krijgen met antibiotica. Dit betreft een onderzoek voor patiënten met een complexe blindedarmontsteking, pas tijdens de operatie zal duidelijk worden of u in aanmerking komt voor deelname. U krijgt nu alvast uitleg over wat het onderzoek inhoudt, zodat u voldoende tijd heeft om te beslissen of u mee wilt doen aan dit onderzoek. Lees deze informatie rustig door en vraag de onderzoeker/arts uitleg als u vragen heeft. U kunt ook de onafhankelijk deskundige, die aan het eind van deze brief genoemd wordt, om aanvullende informatie vragen. En u kunt er ook over praten met uw partner, vrienden of familie. Verdere informatie over meedoen aan zo’n onderzoek staat in de bijgevoegde brochure ‘Medisch-wetenschappelijk onderzoek’.

1. Algemene informatie

Dit onderzoek is opgezet door het Erasmus MC en wordt uitgevoerd door artsen en onderzoekers in verschillende ziekenhuizen in Nederland. Voor dit onderzoek zijn 1066 proefpersonen nodig. De Medisch Ethische Toetsings Commissie van het Erasmus MC heeft dit onderzoek goedgekeurd. Algemene informatie over de toetsing van onderzoek vindt u in de brochure ‘Medisch-wetenschappelijk onderzoek’.

2. Doel van het onderzoek

Het doel van het onderzoek is uitzoeken wat de beste behandelduur van antibiotica is na een operatie voor complexe acute appendicitis. In dit onderzoek willen we nagaan of een kortdurend toedieningsschema (twee dagen) even veilig en effectief is als het standaard schema (vijf dagen).

3. Achtergrond van het onderzoek

Ca. 16.000 patiënten ondergaan jaarlijks in Nederland een appendectomie. In 20 tot 30% daarvan is er sprake van complexe appendicitis. Patiënten met complexe appendicitis hebben een verhoogd risico op een infectieuze complicatie (zoals een abces in de buikholte of een ontsteking van de wond) ten opzichte van patiënten met een simpele appendicitis. Om dit risico te verlagen, krijgen patiënten nog enkele dagen antibiotica toegediend via een infuus. Op dit moment is er geen overeenstemming over de benodigde behandelduur na de operatie en varieert het hoe lang patiënten nabehandeling krijgen. Uit eerder onderzoek komen aanwijzingen dat kortere duur behandeling met antibiotica even veilig en effectief is als de standaard vijf dagen.
Een gevolg van overbehandeling met antibiotica is resistentie van bacteriën tegen antibiotica. Dit is wereldwijd een groeiend probleem. Om het risico op bacteriële resistentie te minimaliseren en gelijktijdig mogelijk opnameduur en zorgkosten omlaag te brengen, is het van belang om te onderzoeken of een kortere behandeling met antibiotica ook veilig en effectief is.

4. Wat meedoen inhoudt

Als u meedoet, duurt dat in totaal ongeveer drie maanden voor u.

Geschiktheidsonderzoek

Eerst bepalen we of u kunt meedoen. U kunt meedoen wanneer u een appendectomie hebt ondergaan waarbij de operateur tijdens de operatie een complexe appendicitis heeft vastgesteld. Wanneer u te ernstig ziek was voorafgaand aan de operatie en/of wanneer de ontsteking in buikholte te uitgebreid was, kunt u niet meedoen in het onderzoek. Wanneer u een bekende allergie heeft voor de antibiotica, kunt u ook niet deelnemen.

Behandeling

Wanneer u geschikt bent bevonden voor deelname aan het onderzoek, gaan we u behandelen met antibiotica. De antibiotica die in dit onderzoek gebruikt worden (cefuroxim en metronidazol via infuus) zijn in Nederland al geregistreerd voor complexe acute appendicitis. Dit betekent dat men al heeft bewezen dat de middelen veilig zijn en werken bij complexe acute appendicitis. Ze worden al decennia voor deze indicatie gebruikt.

U krijgt in de eerste 48 uur na de operatie antibiotica toegediend via een infuus, ongeacht of u wel of niet deelneemt aan de studie. Dit is niet afwijkend van de standaardbehandeling. Om te beoordelen of een kortere behandelduur even goed is als de standaard behandelduur, worden de deelnemers aan het onderzoek ingedeeld in 2 groepen. . De helft van de

proefpersonen krijgt na de eerste 48 uur geen antibiotica meer, de andere helft krijgt nog 3 dagen langer antibiotica via het infuus. Om de verdeling zo correct mogelijk te houden, wordt door loting bepaald in welke groep u komt. De loting vindt plaats uiterlijk 24 uur na uw operatie. Uw behandelend arts en de onderzoekers hebben geen invloed op de uitslag van de loting.

Bezoeken en metingen

Na de opname in het ziekenhuis komt u één keer naar het ziekenhuis terug voor een controle afspraak op de polikliniek Chirurgie, ongeveer twee tot vier weken na de operatie. Dit betreft een standaardcontrole die u ook zal hebben wanneer u niet deelneemt aan het onderzoek. Na ontslag krijgt u via de post of via email een vragenlijst toegestuurd, met het verzoek om deze vier weken na de operatie in te vullen. De vragen kunnen mogelijk persoonlijk of confronterend zijn. Het invullen kost ongeveer tien minuten. Tot slot wordt u drie maanden na de operatie nog één keer thuis opgebeld door de onderzoeker. U krijgt dan vragen over hoe het met u gaat en hoe het herstel na de operatie is verlopen. Het telefoongesprek zal ongeveer vijf tot tien minuten van uw tijd in beslag nemen.

5. Wat wordt er van u verwacht

Als u besluit deel te nemen aan de studie vragen we u om de gemaakte afspraken zo goed mogelijk na te komen, om de instructies zo goed mogelijk op te volgen en om de arts op de hoogte te houden van alle veranderingen in uw gezondheidstoestand. Er gelden geen speciale leefregels of beperkingen wanneer u deelneemt.

De afspraken zijn dat u:

- niet ook nog aan een ander medisch-wetenschappelijk onderzoek meedoet.
- toediening van de antibiotica toelaat volgens de uitleg
- afspraken voor controle (poliklinisch en telefonisch) nakomt.
- de vragenlijst invult en retourneert

Het is belangrijk dat u contact opneemt met de onderzoeker:

- als u in een ziekenhuis wordt opgenomen of behandeld.
- als u plotseling gezondheidsklachten krijgt.
- als u niet meer wilt meedoen aan het onderzoek.
- als uw contactgegevens wijzigen.

6. Mogelijke nadelige effecten

De te onderzoeken behandeling, de kortere behandelduur met antibiotica, zou als nadelig effect kunnen hebben dat u een hogere kans op een infectieuze complicatie hebt. Een infectieuze complicatie zoals een abces in de buikholte is goed te behandelen, maar zou kunnen betekenen dat u langer in het ziekenhuis moet blijven.

7. Mogelijke voor- en nadelen

Het is belangrijk dat u de mogelijke voor- en nadelen goed afweegt voordat u besluit mee te doen. Een mogelijk voordeel is dat de te onderzoeken behandeling zou kunnen zorgen dat uw opnameduur na de operatie korter is, zodat u sneller naar huis kunt om verder te herstellen. Zeker is dit echter niet. Een nadeel van de te onderzoeken behandeling kan zijn, zoals genoemd bij 6, dat u mogelijk een hogere kans zou hebben op een infectieuze complicatie (wanneer u in deze groep geloot wordt). Deelname aan het onderzoek betekent ook dat u wat extra tijd kwijt bent vanwege het invullen van de vragenlijst en de telefonische controle. Al deze zaken zijn hiervoor onder punt 4, 5 en 6 beschreven.

8. Als u niet wilt meedoen of wilt stoppen met het onderzoek

U beslist zelf of u meedoet aan het onderzoek. Deelname is vrijwillig. Als u niet wilt meedoen, wordt u op de gebruikelijke manier behandeld voor uw complexe blindedarmontsteking, namelijk met drie tot vijf dagen antibiotica via infuus na de operatie afhankelijk van het ziekenhuis waar u geopereerd wordt.

Als u wel meedoet, kunt u zich altijd bedenken en toch stoppen, ook tijdens het onderzoek. U wordt dan weer op de gebruikelijke manier behandeld voor uw complexe blindedarmontsteking. U hoeft niet te zeggen waarom u stopt. Wel moet u dit direct melden aan de onderzoeker. De gegevens die tot dat moment zijn verzameld, worden gebruikt voor het onderzoek.

Als er nieuwe informatie over het onderzoek is die belangrijk voor u is, laat de onderzoeker dit aan u weten. U wordt dan gevraagd of u blijft meedoen.

9. Einde van het onderzoek

Uw deelname aan het onderzoek stopt als:

- alle bezoeken zoals beschreven onder punt 4 voorbij zijn
- u zelf kiest om te stoppen
- de onderzoeker het beter voor u vindt om te stoppen
- Het Erasmus MC, de overheid of de beoordelende medisch-ethische toetsingscommissie, besluit om het onderzoek te stoppen.

Het hele onderzoek is afgelopen als alle deelnemers klaar zijn.
Na het verwerken van alle gegevens informeert de onderzoeker u over de belangrijkste uitkomsten van het onderzoek. Dit gebeurt ongeveer binnen 1 jaar na uw deelname.

10. Gebruik en bewaren van uw gegevens

Voor dit onderzoek is het nodig dat uw medische en persoonsgegevens worden verzameld en gebruikt. Elke proefpersoon krijgt een code die aan de gegevens worden gekoppeld. Uw naam en andere persoonsgegevens worden weggelaten.

Uw gegevens

In de algemene brochure is uitgelegd dat de onderzoeker gegevens over u verzamelt en deze vertrouwelijk behandelt. Dit betekent dat een aantal personen uw medische status en de gegevens van het onderzoek mogen inzien. Deze personen mogen de gegevens gebruiken voor dit onderzoek, maar zij mogen de gegevens alleen bekend maken zonder daarbij uw naam of andere persoonlijke gegevens te vermelden. Uw identiteit blijft dus altijd geheim. De onderzoeker bewaart de gegevens met een code. Dit betekent dat op de studie-documenten in plaats van uw naam alleen een letter-cijfercode staat. Alleen de onderzoeker houdt een lijst bij waarop staat welke letter-cijfercode bij welke naam hoort.

Normaal gesproken heeft alleen uw behandelend arts en zijn/haar team inzage in uw gegevens. Als u meedoet aan deze studie krijgen meer mensen inzage in uw medische gegevens en studiegegevens. De personen die inzage kunnen krijgen in uw gegevens zijn:

- de medewerkers van het onderzoeksteam
- de leden van de toetsingscommissie die de studie heeft goedgekeurd
- de bevoegde medewerkers van de Inspectie voor de Gezondheidszorg
- de veiligheidscommissie die het onderzoek in de gaten houdt

Bovengenoemde personen houden uw gegevens geheim. Als u de toestemmingsverklaring ondertekent, geeft u toestemming voor het verzamelen, bewaren en inzien van uw medische en persoonsgegevens.

Na de studie worden de gecodeerde gegevens gedurende 15 jaar bewaard. Dit is nodig om alles goed te kunnen controleren. Bovendien willen wij graag uw gegevens gebruiken voor andere onderzoeken die worden uitgevoerd naar blindedarmontsteking. Deze onderzoeken hebben dus eenzelfde doel als het onderzoek waarvoor u nu wordt gevraagd. Het is dus niet zo dat uw gegevens ook zullen worden gebruikt voor onderzoek naar een geheel andere aandoening of een heel ander probleem. Vanzelfsprekend blijft de vertrouwelijkheid die we hierboven hebben beschreven altijd gelden. Vindt u het goed als wij uw gegevens gebruiken? Als u dat niet wilt, respecteren wij dat natuurlijk. U kunt uw keuze op het toestemmingsformulier aangeven.

Een beschrijving van deze klinische studie staat op de website van het Nederlands Trialregister (http://www.trialregister.nl). Deze website bevat geen informatie die herleidbaar is tot u als persoon. Wel kan de website een samenvatting van de resultaten tonen. U heeft toegang tot deze website. U vindt deze studie onder ”APPIC”.

11. Verzekering voor proefpersonen

Voor iedereen die meedoet aan dit onderzoek is een verzekering afgesloten. De verzekering dekt schade door het onderzoek. Niet alle schade is gedekt. In **bijlage B** vindt u meer informatie over de verzekering. Daar staat ook aan wie u schade kunt melden.

12. Informeren huisarts

Wij laten uw huisarts altijd in een brief weten dat u meedoet aan het onderzoek. Dit is voor uw eigen veiligheid. Als u dit niet goed vindt, kunt u niet meedoen aan dit onderzoek. U kunt niet deelnemen aan het onderzoek als u geen huisarts heeft.

13. Geen vergoeding voor meedoen

De studiebehandeling en extra telefonische controle voor het onderzoek kosten u niets.
U wordt niet betaald voor het meedoen aan dit onderzoek.

14. Heeft u vragen?

Indien u tijdens de studie vragen of klachten heeft, vragen wij u contact op te nemen met de onderzoeker of uw behandelend arts. Indien u twijfelt over deelname kunt u een onafhankelijke arts raadplegen, die zelf niet bij het onderzoek betrokken is, maar die wel deskundig is op het gebied van dit onderzoek en uw ziekte. Ook als u voor of tijdens de studie vragen heeft die u liever niet aan de onderzoekers stelt, kunt u contact opnemen met de onafhankelijke arts. Als u niet tevreden bent over het onderzoek of de behandeling kunt u terecht bij de onafhankelijke klachtencommissie van het Erasmus MC.

De bereikbaarheidsgegevens vindt u in **bijlage A**.

15. Ondertekening toestemmingsformulier

Wanneer u voldoende bedenktijd heeft gehad, wordt u gevraagd te beslissen over deelname aan dit onderzoek. Indien u toestemming geeft, zullen wij u vragen deze op de bijbehorende toestemmingsverklaring schriftelijk te bevestigen. Door uw schriftelijke toestemming geeft u aan dat u de informatie heeft begrepen en instemt met deelname aan het onderzoek.
Het handtekeningenblad wordt door uw behandelend arts bewaard. U krijgt een kopie of een tweede exemplaar van deze toestemmingsverklaring.

Dank voor uw aandacht.

Bijlagen bij deze informatie

A. Contactgegevens

B. Informatie over de verzekering

C. Schema onderzoekshandelingen

D. Toestemmingsformulier proefpersoon

E. Brochure ‘Medisch-wetenschappelijk onderzoek. Algemene informatie voor de proefpersoon’ (versie 01-09-2014)

Bijlage A: contactgegevens voor Erasmus MC

Coördinerend onderzoeker: Drs. E.M.L. de Wijkerslooth

Afdeling Heelkunde, Erasmus MC
's-Gravendijkwal 230
3015 CE Rotterdam
Tel: 010-7034519, 06-17616440

Hoofdonderzoekers: Drs. A.L. van den Boom & Dr. B.P.L. Wijnhoven

Afdeling Heelkunde, Erasmus MC
's-Gravendijkwal 230
3015 CE Rotterdam
Tel: 010-7033727

Onafhankelijk arts: Dr. J. De Jonge, chirurg, Erasmus MC

Afdeling Heelkunde, Erasmus MC
's-Gravendijkwal 230
3015 CE Rotterdam
Tel: 010-7033727

Klachten: klachtencommissie Erasmus MC - 010 703 3198

Bijlage B: informatie over de verzekering

Voor iedereen die meedoet aan dit onderzoek, heeft het Erasmus MC een verzekering afgesloten. De verzekering dekt schade door deelname aan het onderzoek. Dit geldt voor schade tijdens het onderzoek of binnen vier jaar na het einde ervan. Schade moet u binnen die vier jaar aan de verzekeraar hebben gemeld.

De verzekering dekt niet alle schade. Onderaan deze tekst staat in het kort welke schade niet wordt gedekt. Deze bepalingen staan in het Besluit verplichte verzekering bij medisch-wetenschappelijk onderzoek met mensen. Dit besluit staat op www.ccmo.nl, de website van de Centrale Commissie Mensgebonden Onderzoek (zie 'Bibliotheek' en dan 'Wet- en regelgeving').

Bij schade kunt u direct contact leggen met de verzekeraar:

De verzekeraar van het onderzoek is:	
Naam:	CNA Insurance Company Limited
Adres:	Strawinskylaan 703, 1077 XX Amsterdam
Telefoonnummer:	+31 (0) 20 573 7274
E-mail:	esther.vanherk@cnahardy.com
Contactpersoon:	Mw. Esther van Herk

De verzekering biedt een dekking van € 650.000 per proefpersoon en € 5.000.000 voor het hele onderzoek (en € 7.500.000 per jaar voor alle onderzoeken van dezelfde opdrachtgever).

De verzekering dekt de volgende schade **niet**:

- schade door een risico waarover u in de schriftelijke informatie bent ingelicht. Dit geldt niet als het risico zich ernstiger voordoet dan was voorzien of als het risico heel onwaarschijnlijk was;
- schade aan uw gezondheid die ook zou zijn ontstaan als u niet aan het onderzoek had meegedaan;
- schade door het niet (volledig) opvolgen van aanwijzingen of instructies;
- schade aan uw nakomelingen, als gevolg van een negatief effect van het onderzoek op u of uw nakomelingen;
- schade door een bestaande behandelmethode bij onderzoek naar bestaande behandelmethoden.

Bijlage C – Schema onderzoekshandelingen

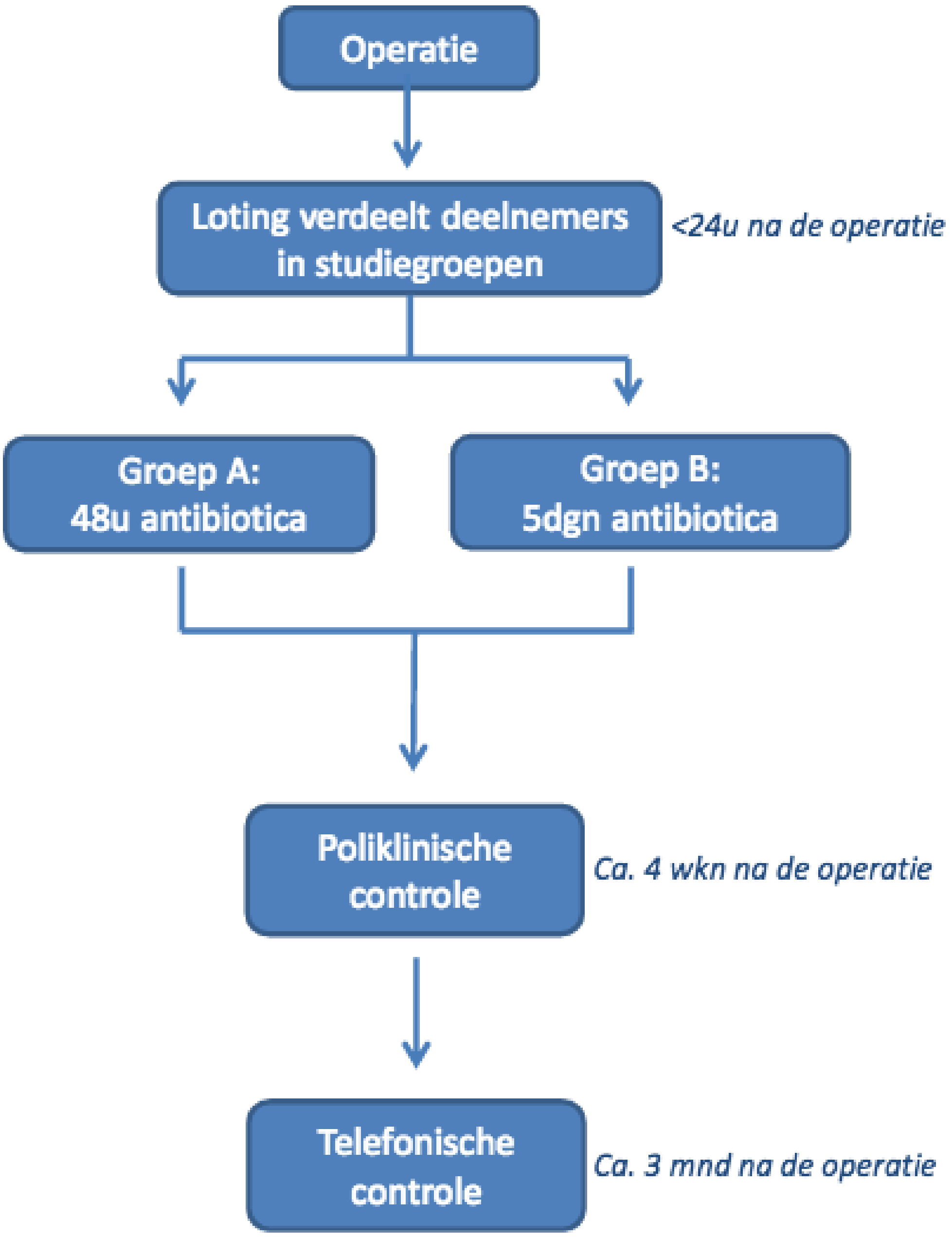

Bijlage D: Toestemmingsformulier proefpersoon

Onderzoek naar effectiviteit van een kortdurende behandeling met postoperatieve antibiotica bij complexe blindedarmontsteking

- Ik heb de informatiebrief gelezen. Ook kon ik vragen stellen. Mijn vragen zijn voldoende beantwoord. Ik had genoeg tijd om te beslissen of ik meedoe.
- Ik weet dat meedoen vrijwillig is. Ook weet ik dat ik op ieder moment kan beslissen om toch niet mee te doen of te stoppen met het onderzoek. Daarvoor hoef ik geen reden te geven.
- Ik geef toestemming voor het informeren van mijn huisarts dat ik meedoe aan dit onderzoek.
- Ik weet dat sommige mensen mijn gegevens kunnen inzien. Die mensen staan vermeld in deze informatiebrief.
- Ik geef toestemming voor het verzamelen en gebruiken van mijn gegevens op de manier en voor de doelen die in de informatiebrief staan.
- Ik geef toestemming om mijn gegevens op de onderzoekslocatie nog 15 jaar na dit onderzoek te bewaren.
- Ik geef □ **wel**
  □ **geen** toestemming om mij na dit onderzoek opnieuw te benaderen voor een vervolgonderzoek.
- Ik wil meedoen aan dit onderzoek.

Naam proefpersoon:
Handtekening: Datum : __ / __ / __

Ik verklaar dat ik deze proefpersoon volledig heb geïnformeerd over het genoemde onderzoek.

Als er tijdens het onderzoek informatie bekend wordt die de toestemming van de proefpersoon zou kunnen beïnvloeden, dan breng ik hem/haar daarvan tijdig op de hoogte.

Naam onderzoeker (of diens vertegenwoordiger):
Handtekening: Datum: __ / __ / __

<indien van toepassing>
Aanvullende informatie is gegeven door:
Naam:
Functie:
Handtekening: Datum: __ / __ / __

De proefpersoon krijgt een volledige informatiebrief mee, samen met een kopie van het getekende toestemmingsformulier.
